# Supplementary material for: Optofluidic memory and self-induced nonlinear optical phase change for reservoir computing in silicon photonics
Source: Nat Commun. 2023 Jul 21;14:4421. doi: 10.1038/s41467-023-40127-x (PMC10362060; doi:10.1038/s41467-023-40127-x)
Supplement: Supplementary file 1 — Supplementary Information [file 41467_2023_40127_MOESM1_ESM.pdf]

SUPPLEMENTAL MATERIAL for:

Optofluidic memory and self-induced nonlinear optical phase change for reservoir computing in silicon photonics

Chengkuan Gao[1], Prabhav Gaur[1], Dhaifallah Almutairi[1,2], Shimon Rubin[1] and Yeshaiahu Fainman[1]

[1] *Department of Electrical and Computer Engineering, University of California, San Diego, 9500 Gilman Dr., La Jolla, California 92023, USA*

[2]*King Abdulaziz City for Science and Technology (KACST), P.O. Box 6086, Riyadh 11442, Saudi Arabia*

S.1. OUTPUT PORTS DISTANCE AND FRINGE GENERATION ON CCD CAMERA

The relation between the shift of the objective from the image plane L, wavelength λ, the distance between two output ports d, and the fringe width W, can be determined by calculating the optical path difference in free space, given by

$$W = L\lambda/d. \tag{S1}$$

By choosing $d = 20$ μm, $L \simeq 300$ μm, $\lambda = 1550$ nm, the corresponding fringe width on the front focal plane is $W \simeq 24$ μm. Since the objective and the lens form a 4f imaging system with 125 times magnification, the fringe occupies 125×24 $\mu m \simeq 3$ mm on the CCD sensor. The latter admits horizontal dimension of length 9.6 mm and therefore assuming the parameters above, the sensor can accommodate three fringes, providing sufficient information on the fringes shift during the self-induced phase change.

S.2. ELECTROSTATIC DEPOSITION RATE AS A FUNCTION OF TIP-CHIP DISTANCE

Here we provide direct evidence that the origin of silicone oil droplets deposition mechanism from glass tip into the chip is based on electrostatic forces. In our experience emission rate of silicone oil droplets does not change appreciably during one hour time scale. With this in mind, we can assume that after the electrostatic charge on the glass tip is induced by the triboelectric effect, the charge does not change it time during the experiment and we expect that the silicone oil on the tip should experience stronger attraction force to the chip if the tip-chip distance is reduced. Indeed, Fig.S1 below

demonstrates that for some arbitrary initial location of the glass tip (estimated to be few tens of μm above the chip), further shift of the tip away from the chip decreases the deposition rate and increases the time needed to fill the cell.

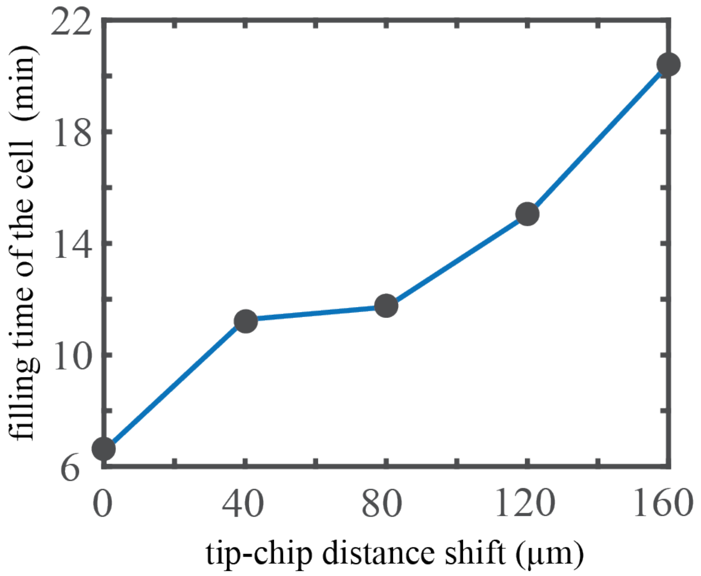

FIG. S1. Experimental result indicating that tip-chip distance increase leads to increase of cell's filling time and hence to decrease of the deposition rate. In turn it signals that the origin of the deposition rate is electrostatic-based. The initial tip-chip distance is unknown and estimated to be few tens of μm.

S.3. EXTRACTION OF OPTICAL PHASE CHANGE AND NOISE ANALYSIS

As mentioned above, in our experiments we detect the self-induced phase change effect due to liquid film deformation, by monitoring shift of the interference fringes. In order to determine the noise threshold, i.e., the minimal phase change triggered by modification of some physical parameter, we perform two types of measurements. First, we would like to determine the role of TC effect relative to the more traditional heat-based TO effect by comparing shift of interference fringes in case the liquid cell is filled with silicone oil to the case when the liquid cell remains empty, presented in Fig.S2a and Fig.S2b, respectively. In particular, Fig.S2 presents typical data used in Fig.2, where solid curve is the raw acquired data obtained by taking the arithmetic mean of all rows, whereas the dashed curve is generated by applying the additional step of MATLAB's

built-in 'smooth' function. Assuming that the distance between two adjacent peaks in the same curve is 2π, fringe shift due to TC-effect in case of 500 nm thick silicone oil is $2\pi \cdot 157/195 = 1.61\pi$. Similar measurement without liquid film presented in Fig.S2b presents phase shift of 7 pixels. Since our field of view captures about three fringes and each fringe occupies approximately 200 pixels, the minimal resolution of the phase change measurement is around $2\pi/200 = 0.01\pi$ rad.

To understand whether the 7 pixels shift presented in Fig.S2b is below or above noise threshold we perform second type of measurement presented in Fig.S2c where active WG without liquid is exposed to oscillatory or constant optical power. The acquired data for the case of constant power described by central and bottom panels of Fig.S2c) over a span of 5 s for power levels 1 mW and 22 mW, respectively, indicate that the acquired signal is subject to about two pixels natural fluctuations presumably due to mechanical vibrations, corresponding to 0.02π rad phase shift.

For a device without liquid film, there should be no fringe movements apart from the random noise, but as shown in Fig.S1c1, when doing a 1 Hz modulation between 1 mW and 22 mW without liquid film, around 7 pixels modulation depth is also monitored, which is not random and much higher than 2 pixels. This fringe movement may come from two parts, first is when changing the light intensity, the imperfection of our imaging system will shift the intensity distribution a little bit, causing some slight movement of the fringe peak location, as shown in Fig.S2b which plots the raw and smoothed fringe curve, we can see that from blue curve to red curve, the left part of each fringe still matches, the fringes are more expanded rather than moved. The second possibility is thermal optical effect induced phase change, we will discuss this part in the following sections.

S.4. ESTIMATION OF OPTICAL POWER IN THE ACTIVE/PASSIVE WAVEGUIDE

To estimate the optical power in the active/passive WG we consider several loss mechanisms. Based on our measurement results in a single bare WG without liquid cell and without gold patch, under best fiber-chip coupling condition, 1 mW input laser power (before coupling into the chip) yields 50 μW after the 0.42 NA objective lens, which collects the light in a light cone with a $\sim 50^o$ apex angle, as shown by the red curve

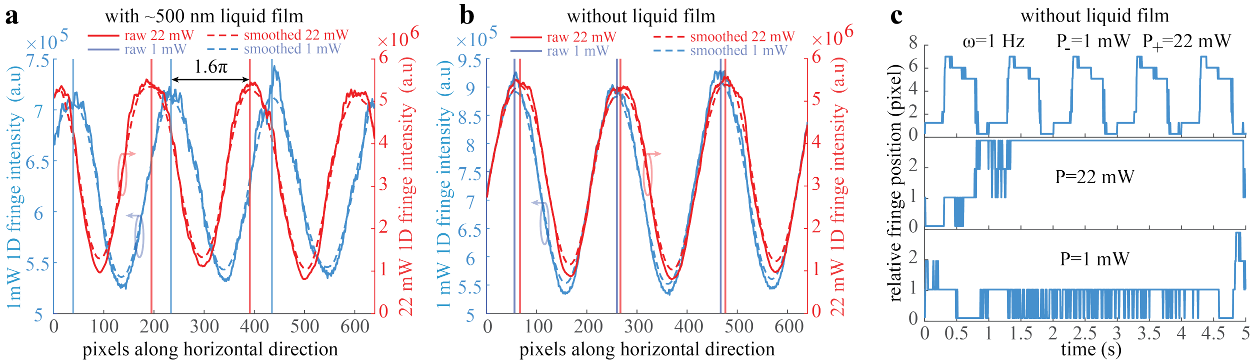

FIG. S2. Experimental results analyzing: (a) interference fringe shift due to power increase from 1 mW to 22 mW, as a result of 500 nm thick silicon oil film deformation. (b) Reference case against (a) but with empty trench without silicone oil. (c) Location of single fringe peak as a function of time in case of 1 Hz input signal modulation (top panel), constant 22 mW (central panel), and constant 1 mW (bottom panel) all occurring without presence of liquid film.

in Fig.S3. By a frequency domain wave optics COMSOL simulation which simulates WG emits TM polarized light into free space, we can get the optical power intensity distribution as shown in the color map of Fig.S3. From this simulation, approximately 28% of optical energy sits inside the light cone. Furthermore, the transmittance of the objective is 60% @1550 nm, the impedance mismatch for TM mode (effective index 1.7) yields another 10% loss, hence combining with the above, and assuming negligible propagation loss in silicon WG, we estimate fiber-to-chip insertion loss to be $10\cdot\log(50/(1000\cdot 0.9\cdot 0.28\cdot 0.6))\simeq -5$ dB. Consequently, the WG power P_{WG} can be related to input power P_I via $P_{WG}=P_I/6$ where additional factor of two stems from splitting of the input WG into two WGs.

S.5. COMPARISON OF SELF-INDUCED PHASE CHANGE DUE TO THERMOCAPILLARY EFFECT AND TO THERMO-OPTICAL EFFECT

After having a rough estimation of in-waveguide power, we employed COMSOL in order to simulate the TO effect in a single WG. In particular, we utilized computational scheme similar to the scheme schematically described in Fig.2a, where the liquid was replaced with air and TO effect stems from refractive index changes of silicon; TO coefficient $dn/dT = 1.8\cdot 10^{-4}$ 1/K. The corresponding phase change due to TO effect

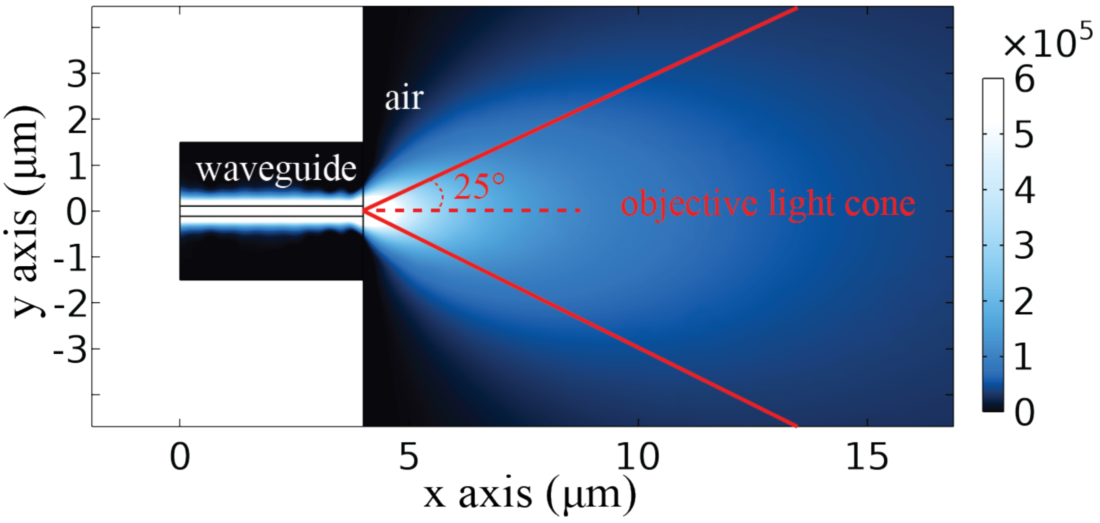

FIG. S3. Optical simulation result presenting optical mode emitted from WG into the free space, and the spatial region corresponding to NA of the objective collecting the optical power. The latter is approximately equal to 28 %.

of Si is presented in Fig.S4. For a case when the input power is 22 mW, based on our estimate above the corresponding in-WG power is $22/6 \simeq 4$ mW, and according to Fig.S4 it yields phase change $\simeq 0.06\pi$ rad. The latter admits the same order of magnitude as the measured 7 pixels fringe movement from 1 mW to 22 mW input power. Given all the 7 pixel shift comes from the TO effect, the TC effect is still 1.7/0.7 $\simeq$ 24 times larger than the TO effect.

S.6. PHASE MODULATION UNDER COMBINED DROP-BY-DROP DEPOSITION AND INPUT POWER MODULATION

Here we present complementary measurement of the self-induced phase change effect, as well as the nonlinear response ΔP_{nl} under fixed driving frequency of 10 Hz and increasing total liquid volume, enabling to measure the nonlinear response $\Delta\varphi$ as a function of liquid thickness. Instrumental to conduct this measurement is drop-by-drop electrostatic-based deposition enabling to deliver silicone oil droplets of volume of a few femtoliters. Fig.S5a presents the effect of continuous droplet deposition into initially empty liquid cell at rate of $\delta = 1.7$ droplets per second, affecting the phase change between the active and the passive WGs. As expected, the maximal and the minimal

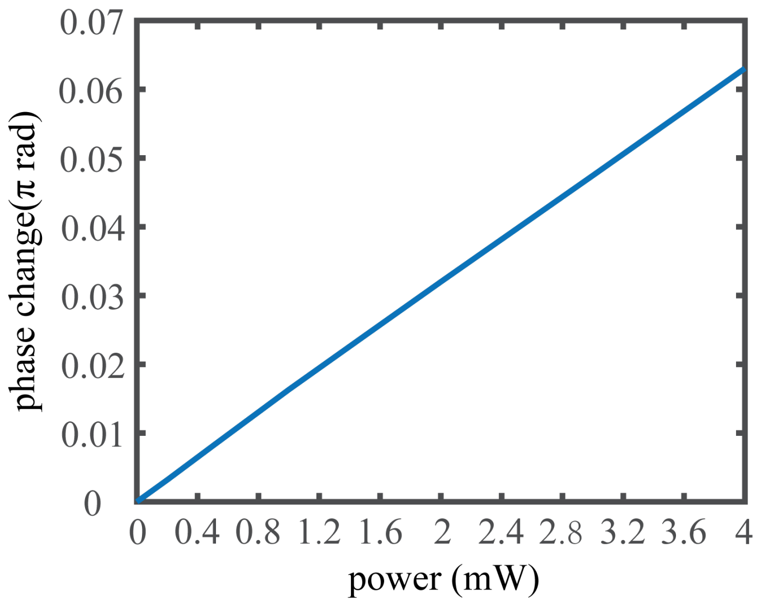

FIG. S4. Numerical simulation results providing phase change as a function of optical power in the active WG due to TO effect of silicon. The corresponding computational domain is identical to the scheme provided in Fig.2a but without the liquid film.

phase change values of the envelope, φ_+ and φ_-, respectively, are both strictly increasing functions of time due to monotonically increasing liquid thickness. However, the phase modulation at each time step defined as

$$\Delta\varphi = \varphi_+ - \varphi_-, \tag{S2}$$

admits vanishing values at early and late times, which corresponds to thin and thick liquid film configurations, both admitting negligible response. The maximal response value is attained at ~ 160 s which corresponds to ~ 300 nm thick liquid film. Fig.S5b presents phase change evolution in the interval $140 - 150$ s, allowing to distinguish between successive droplet deposition events separated by time δt, where each droplet modifies φ_+ and φ_- by a discrete step.

S.7. XOR PERFORMANCE FOR LONGER RELAXATION TIMES

Fig.S6 presents performance of XOR task for $\tau_w = 50$ ms but longer relaxation times $\tau_r = 40, 70$ ms.

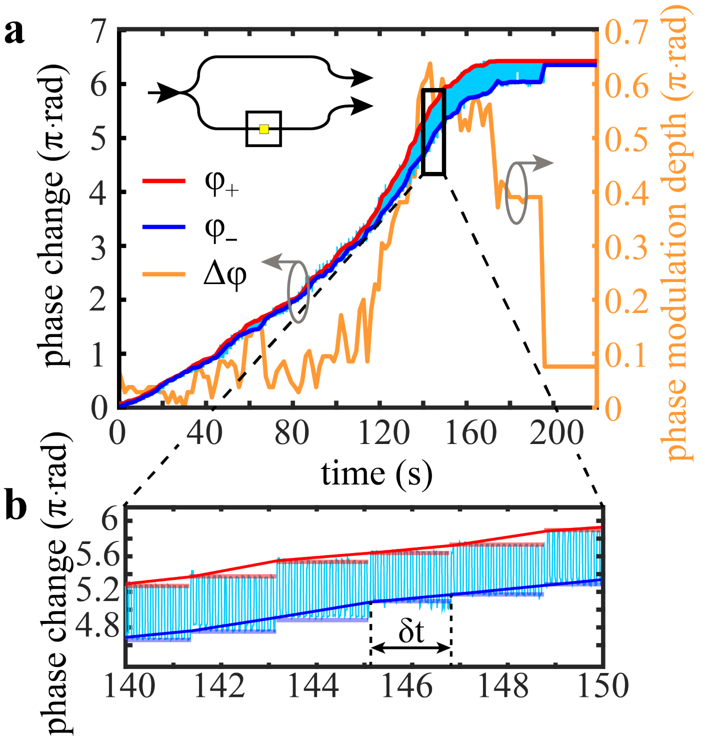

FIG. S5. Self-induced phase change effect under drop-by-drop deposition and periodic actuation. **a**, The envelopes of the self-induced phase change $\varphi_{\pm}$ and the corresponding difference $\Delta\varphi$ (defined by Eq.S2), plotted along the vertical left and right axes, respectively. **b**, Magnified region in **a** between 140-150 s, demonstrating increase of φ_{+} and φ_{-} values after discrete droplet deposition approximately at frequency $1/\delta t$. See Supplementary Movie 4 for demonstration of drop-by-drop deposition combined with optical modulation.

S.8. NUMERICAL SIMULATION: NARMA2 COMPUTATION AS A FUNCTION OF NUMBER OF LIQUID CELLS AND NUMBER OF INPUTS IN THE RESERVOIR

Fig.S7 presents numerical simulation result of RC of NARMA2 task as a function of number of liquid cells and the number of inputs/outputs (reservoir size) in the photonic network. The number of time steps used for training is 400 whereas for testing is 100 throughout the considered cases with maximal power 0.1 mW. Fig.S7a presents RC results with an MZI hosting a single liquid cell in one of the arms, demonstrating very good agreement between the predicted and the actual signals with average NMSE value 0.0023. Fig.S7b presents a schematic description of photonic WGs network where each intersection is a symmetric directional coupler, and each rectangle is a liquid cell. In such

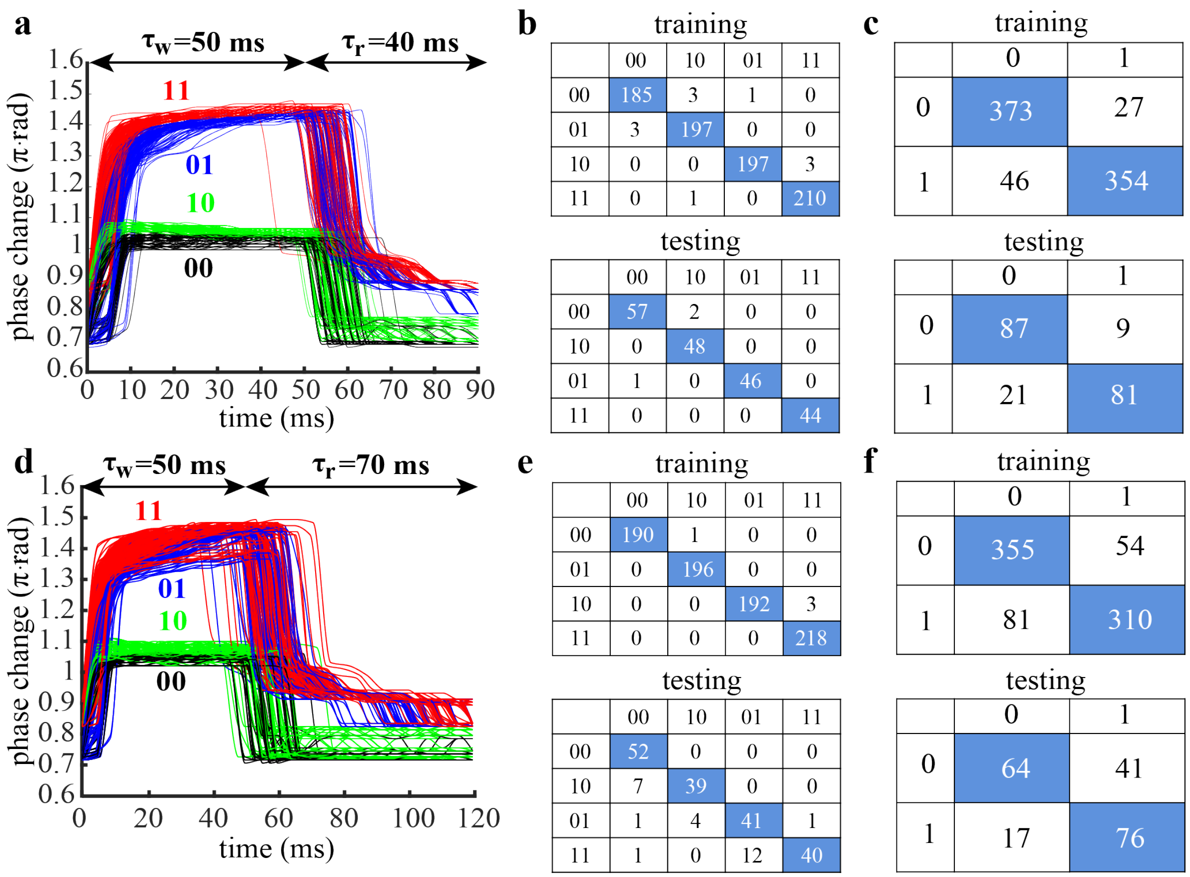

FIG. S6. **RC-based classification of 2D and 4D task for $\tau_r = 40, 70$ ms, complementing the $\tau_r = 10$ ms case presented in the main text. a,d**, Folded dynamics plot of pulses duration $\tau_w = 50$ ms and relaxation time $\tau_r = 40, 70$ ms. **b,e**, Confusion matrices of the 00, 01, 10, 11 classification for $\tau_r = 40, 70$ ms where the vertical axis corresponds to actual (input) values whereas the horizontal axis corresponds to predicted (output) values. **c,f**, Confusion matrix of 2D classification for $\tau_r = 40, 70$ ms.

architecture the number of layers is equal to the number of inputs/outputs (reservoir size), and facilitates signal mixing from all input WGs prior to arrival to the output layer. Fig.S7c presents NMSE as a function of reservoir size due to RC performed in circuits described in Fig.S7b (for which the reservoir size is 8). In particular, reservoir size 0 indicates linear regression-based performance without utilizing the reservoir, hence indicating that employing reservoir size 2 already improves the performance by a factor of ~ 2.5, whereas naively increasing the reservoir size does not lead to significant improvement for the specified values. The latter suggests that further optimization could

be made, and in fact Fig.S7d presents NMSE values as a function of number of liquid cells in a reservoir presented Fig.S7b, where the liquid cells are positioned randomly across the photonic circuit. Interestingly, increasing the number of cells beyond 8 does not significantly improve the performance. It is worth mentioning that in the studied optofluidic system the most dominant short-term memory is one step back in time, and hence computation accuracy reduces as a function NARMA order. For completeness, NMSE values of testing stage for NARMA2, NARMA3 and NARMA 4 tasks are 0.0023, 0.0485 and 0.0596, respectively, whereas the corresponding NMSE values obtained by implementing linear regression only are given by 0.0054, 0.063 and 0.0595.

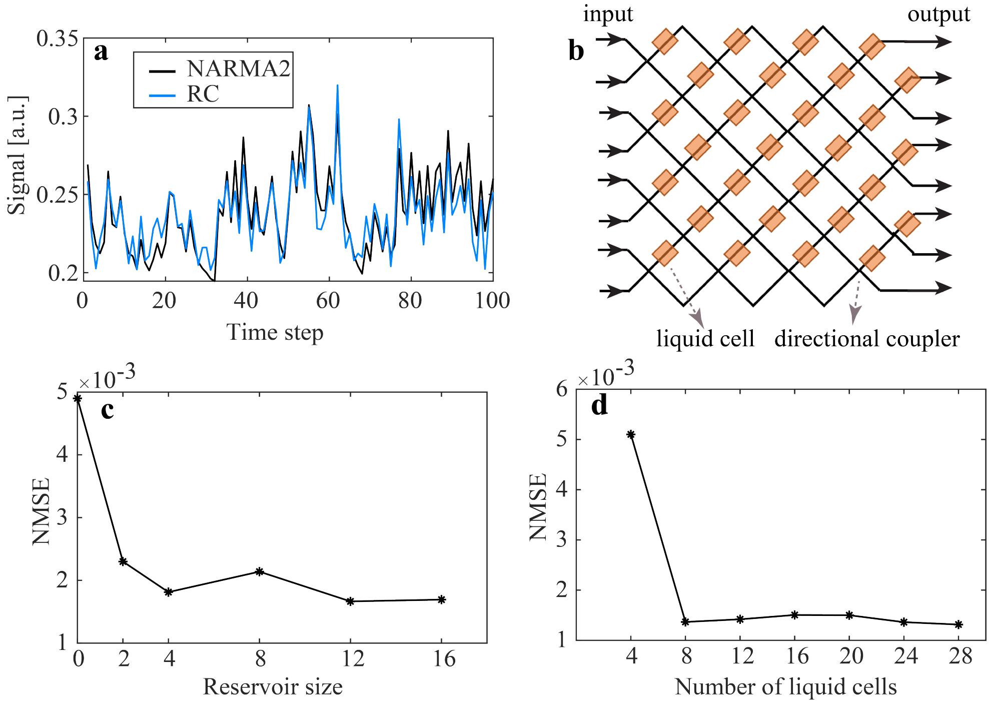

FIG. S7. Numerical results demonstrating RC of NARMA2 task using optofluidic circuits as a function of number of inputs/outputs as well as number of liquid cells. **a** Comparison between RC result and the actual values demonstrating low NMSE value 0.0023. **b** Typical architecture of photonic circuits used to study NARMA2 performance as a function of reservoir size and number of liquid cells. **c** NMSE as a function of reservoir size (number of input/output WGs), demonstrating improvement compared to the case without reservoir (labeled as '0'). In all cases only single liquid cell is used. **d** NMSE as a function of number of liquid cells in a network with a fixed number of eight inputs/outputs, and placing liquid cells in random position.

S.9. EXPERIMENTAL RC-BASED HANDWRITTEN DIGITS RECOGNITION

Fig.S8 presents experimental results of handwritten $0-9$ digits recognition (MNIST data set) using MZI with a single input and a liquid cell embedded in one of its arms, and a single output. In our experiments we down sampled the image from 28×28 to 14×14 to ensure that fiber-chip coupling does not change during the computation time (see Fig.S8a for representative images). Fig.S8b a typical encoded signal injected to the reservoir, implementing row by row image injection, as well as response signal after it passed through the reservoir. The corresponding confusion matrix summarizing RC classification of handwritten $0-9$ digits is given in Fig.S8c naturally indicating better performance for more distinct digits (e.g., 0 and 1) and high error values (encoded as % in the colorbar) for less distinct digits (e.g., 4 and 9).

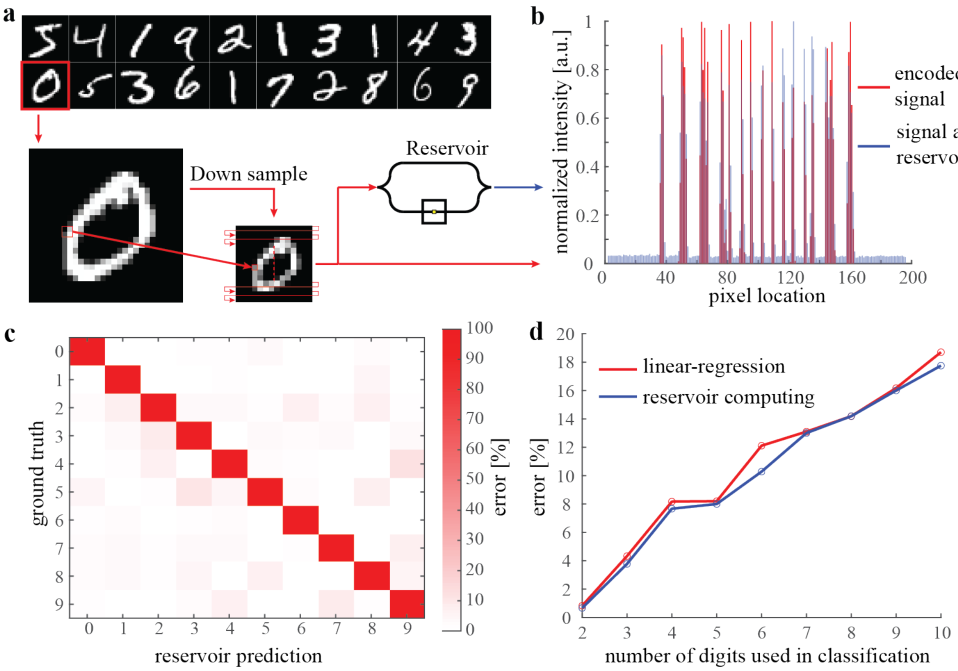

FIG. S8. Experimental results demonstrating RC of MNIST task using optofluidic MZI. **a** Sample of the 28 × 28 digit images that were down-sampled to 14 × 14 images (see methods section) and subsequently injected into the optofluidic MZI circuit with one of the arms. (b) Typical encoded input signal and the resultant output signal after the reservoir of a single raw in one of the digits. (c) Confusion matrix for 0-9 digits classification with colorbar encoding the corresponding error. (d) Performance as a function of number of digits, demonstrating lower error by using reservoir compared to linear regression.
