# Supplementary material for: Optofluidic memory and self-induced nonlinear optical phase change for reservoir computing in silicon photonics
Source: Nat Commun. 2023 Jul 21;14:4421. doi: 10.1038/s41467-023-40127-x (PMC10362060; doi:10.1038/s41467-023-40127-x)
Supplement: Supplementary file 3 — Description of Additional Supplementary Files [file 41467_2023_40127_MOESM3_ESM.pdf]

Description of Additional Supplementary Files

Supplementary Movie 1: presents preparatory step of drop-by-drop deposition of silicone oil femtoliter droplets into the liquid cell, allowing to deposit optically thin liquid film above the Si WG.

Supplementary Movie 2: presents top view microscopy image of the 0.5µm thick liquid film undergoing TC-driven deformation and surface tension driven relaxation, as well as fringes shift serving as a direct indication of self-induced phase change.

Supplementary Movie 3: presents top view microscopy image of the 1 µm thick liquid film undergoing TC-driven deformation and surface tension driven relaxation, as well as fringes shift serving as a direct indication of self-induced phase change.

Supplementary Movie 4: presents top view microscopy image of the liquid film undergoing TCdriven deformation and under 50 ms long pulse and subsequent surface tension driven relaxation during 10 ms time period, which was used in performing XOR task described in Fig.4a.

Supplementary Movie 5: presents the combined effect of drop-by-drop deposition and square wave modulation, as described in Fig.S5.
